# Regional MRI Perfusion Measures Predict Motor/Executive Function in Patients with Clinically Isolated Syndrome

**DOI:** 10.1155/2014/252419

**Published:** 2014-01-23

**Authors:** Efrosini Z. Papadaki, Panagiotis G. Simos, Vasileios C. Mastorodemos, Theodora Panou, Thomas G. Maris, Apostolos H. Karantanas, Andreas Plaitakis

**Affiliations:** ^1^Department of Radiology, Faculty of Medicine, University of Crete, University Hospital, Heraklion, 71110 Stavrakia, Greece; ^2^Department of Medical Imaging MRI Unit, University Hospital, Heraklion, 71110 Stavrakia, Greece; ^3^Department of Psychiatry, Faculty of Medicine, University of Crete, University Hospital, Heraklion, 71110 Stavrakia, Greece; ^4^Department of Neurology, Faculty of Medicine, University of Crete, University Hospital, Heraklion, 71110 Stavrakia, Greece; ^5^Department of Medical Physics, Faculty of Medicine, University of Crete, University Hospital, Heraklion, 71110 Stavrakia, Greece

## Abstract

*Background*. Patients with clinically isolated syndrome (CIS) demonstrate brain hemodynamic changes and also suffer from difficulties in processing speed, memory, and executive functions. *Objective*. To explore whether brain hemodynamic disturbances in CIS patients correlate with executive functions. *Methods*. Thirty CIS patients and forty-three healthy subjects, matched for age, gender, education level, and FSIQ, were administered tests of visuomotor learning and set shifting ability. Cerebral blood volume (CBV), cerebral blood flow (CBF), and mean transit time (MTT) values were estimated in normal-appearing white matter (NAWM) and normal-appearing deep gray Matter (NADGM) structures, using a perfusion MRI technique. *Results*. CIS patients showed significantly elevated reaction time (RT) on both tasks, while their CBV and MTT values were globally increased, probably due to inflammatory vasodilation. Significantly, positive correlation coefficients were found between error rates on the inhibition condition of the visuomotor learning task and CBV values in occipital, periventricular NAWM and both thalami. On the set shifting condition of the respective task significant, positive associations were found between error rates and CBV values in the semioval center and periventricular NAWM bilaterally. *Conclusion*. Impaired executive function in CIS patients correlated positively with elevated regional CBV values thought to reflect inflammatory processes.

## 1. Introduction

Conventional MRI (i.e., T2-weighted, FLAIR, DIR, and pre- and postcontrast T1-weighted images) has been widely used for the assessment and monitoring of patients with multiple sclerosis (MS), due to its high sensitivity in detecting MS-related cerebral white matter (WM) and gray matter (GM) lesions and its ability to quantify their volumes [1]. However, histological studies have revealed pathological damage in WM and GM areas that appeared normal in conventional MRI [2], a finding that is probably related to the poor association between brain total T2 lesion load and degree of physical disability of these patients [3].

On the contrary, nonconventional structural MR imaging (i.e., magnetization transfer imaging, diffusion tensor imaging, MR spectroscopy) proved valuable for assessing the extent of microscopic WM and deep GM damage even in clinically isolated syndrome (CIS), which represents the earliest stage of MS development [1, 4]. Further, hemodynamic changes in both WM and GM may serve as an early sign of regional involvement in disease progression [5]. Dynamic susceptibility-contrast-enhanced T2*-weighted MRI (DSC-MRI) has been used successfully to index regional perfusion [1]. Recent studies have reported a widespread reduction in cerebral blood flow (CBF) in both normal-appearing white matter (NAWM) and normal-appearing deep gray matter (NADGM) in patients with the relapsing-recurring form of MS (RR-MS), indicating diffuse vascular damage and global ischemia [5–12].

There is much less information on the nature of neuronal pathology associated with CIS [13, 14]. The latter is defined by a single acute or subacute episode of neurological disturbance that could be attributed to a WM lesion. A significant proportion of patients with CIS demonstrate cognitive deficits, albeit to a lesser extent than patients with clinically definite MS [15–20]. Impaired memory and attention and reduced speed of processing have been easier to document across studies. There are also some indications of executive deficits on tasks measuring inhibition and set shifting although disentangling executive from attentional and processing speed deficits is not always straightforward (e.g., [18]).

In a recent study, we found widespread increases in cerebral blood volume (CBV) and mean transit time (MTT) values of NAWM and NADGM structures in CIS patients, indicative of postinflammatory changes [5]. These findings are consistent with recent neuropathologic data showing that global inflammation, involving the cerebral cortex, meninges and subcortical brain regions, occurs in patients with early MS [21].

The goal of the present study was to investigate the functional significance of the perfusion changes detected in patients with CIS, by examining the pattern of associations between perfusion indices and performance on tasks designed to isolate executive abilities (response inhibition and set shifting). We hypothesized that in CIS patients perfusion disturbances, indicative of inflammation, are associated with reduced performance on indices of executive dysfunction independent of the well-established processing speed deficits. To test this hypothesis we performed detailed experimental tests designed to assess visuomotor processing speed and specific executive skills in CIS patients and healthy volunteers matched on demographic variables and FSIQ. We used the DSC-MRI technique to estimate perfusion indices on a fine grid of 20 NAWM and NADGM structures bilaterally, including those believed to serve as integral components of brain circuits responsible for visuomotor learning and executive functions, such as the thalamus, basal ganglia, and dorsal frontal regions [22–24]. We then examined whether the detected hemodynamic changes correlated with processing speed *and/or* specific executive abilities in CIS patients.

## 2. Methods and Material

### 2.1. Subjects

CIS patients were recruited through the MS epidemiology program project of Crete and fulfilled the clinical and MRI criteria of the international panel on MS [25]. The CIS group consisted of 30 consecutive patients who had experienced a single neurological episode (initial clinical attack) without having experienced a recurrence up to the time of the MRI study. None of the CIS patients included in the study had received steroids for at least 3 months prior to study inclusion and none of them was on any immunomodulatory treatment. Other exclusion criteria included (a) history of alcohol or drug abuse, head injury with loss of consciousness, schizophrenia or bipolar disorder, learning disability, or any other neurological disorder, (b) autoimmune and/or immune-mediated diseases and infectious diseases, and (c) significant visual or motor impairment that would interfere with executive function.

Given the sensitivity of the neuropsychological data to the cultural background of the subjects and in light of recent epidemiological data from Crete highlighting the impact of environmental factors on MS [26], our CIS patients were compared to 43 healthy volunteers (randomly ascertained from the Cretan population) matched for gender, age, education, FSIQ, and place of residence (urban versus rural).

The study was approved by the University Hospital Ethics Committee and written informed consent was obtained from all patients after being briefed on study details.

### 2.2. Experimental Tasks

Patients and healthy controls were examined on two computerized tasks designed to assess visuomotor processing speed and specific executive skills. Each task was designed to afford contrasts between indices reflecting primarily speed of processing (known to be significantly elevated in CIS patients) and indices reflecting both speed of processing and primarily one executive function domain (response inhibition or cognitive switching).

The visuomotor learning task assessed the ability to learn (Simple condition) and subsequently inhibit a learned manual stimulus-response (S-R) association and adopt a novel S-R association (inhibition condition). During each block of trials, one of two capital letters was flashed at the center of the computer screen at a rate of 1 letter every 1.5 sec and participants had to press the key corresponding to the letter they saw each time. Following a series of 6 learning/habituation trials (simple condition), they were asked to reverse their response strategy and press the “noncorresponding” key for the next 14 trials (inhibition trials). For example, they were first asked to press A if they saw the letter A and Λ if they saw the letter Λ. After the 6th trial they were asked to start pressing the A key in response to the letter Λ and the Λ key in response to the letter A. A different pair of randomly arranged letters was used on each of the four blocks of 20 trials. Number of errors and reaction time (RT) for correct responses was stored electronically and used to compute the following performance indices: (i) average RT across all simple trials, (ii) average RT across all inhibition trials, (iii) mean error rate for simple trials, and (iv) mean error rate for inhibition trials. Only trials with RTs between 200 and 5000 ms (corresponding to ±3 SDs from the group mean of single-trial responses) were included in the computation of the aforementioned indices to eliminate outlier responses.

The Complex Decision task was designed to assess the ability to alternate between two simple cognitive/processing strategies depending on the modality of the stimulus. Auditory (30 animal names) and visual stimuli (30 line drawings of the same animals rendered in one of four single colours: red, blue, green, and yellow) were presented in a pseudorandom order over 116 trials. Visual stimulus presentation time was 1 sec. Participants were instructed to press the right mouse key to respond to (a) spoken words if the named animal had four legs (e.g., *goat, lizard*: auditory targets) and (b) to red animal drawings (of a goat, a lizard, etc.). Stimuli meeting these requirements were considered as targets. Thus, auditory stimuli required semantic processing of the entity corresponding to each word, whereas responding to visual stimuli had to rely merely on visual properties of the stimulus (ignoring the visual or semantic attributes of the depicted object). All other stimuli required a left key press (nontargets). The order of stimuli was designed to afford computation of mean RT and error rates for two experimental conditions: (a) *Simple Processing Trials (SPTs)* which were always preceded by a same-modality stimulus and did not require a shift in either cognitive strategy or responding hand (e.g., /visual target/, /visual target/, /visual target/ [SPT trials are underlined]); and (b) *Strategy and Response Shifting Trials (SRSTs) *requiring a switch in both strategy and responding hand (/visual target/, /auditory nontarget/, /visual target/ [SRST trials underlined]).

### 2.3. Neuropsychological and Psychoemotional Measures

In order to ensure compatibility on FSIQ between the patients and healthy comparison group (in addition to demographic variables), we administered two scales from the Wechsler Abbreviated Scales of Intelligence (WASI), as indices of crystallized (Vocabulary) or fluid intelligence (Matrices) [27].

Psychoemotional variables (depression and anxiety) were also assessed as covariates in the relation between perfusion and executive performance, using the Greek adaptations of the Center for Epidemiology Studies Depression Scale (CES-D) [28] and the State-Trait Anxiety Inventory Form Y [29, 30]. Finally, the fatigue severity scale was administered to all patients in order to assess the severity, frequency, and impact of fatigue on daily life [31].

### 2.4. Magnetic Resonance Imaging Acquisition and Data Analysis

Brain MRI examinations were performed on a clinical 1.5 T whole-body superconducting imaging system (Vision/Sonata, Siemens/Erlangen), equipped with high performance gradients (Gradient strength: 40 mT/m, Slew rate: 200 mT/m/ms) and a two-element circularly polarized head array coil. A basic imaging protocol for multiple sclerosis was applied comprised of a 3D T1-w sequence (MPRAGE, TR 1570/TE 1.73 ms, 160 axial slices), a T2TSE sequence (TR/TE = 5000/98 ms) with contiguous 4 mm thick axial sections, and also a TURBO-FLAIR sequence (TR/TE/TI = 9000/120/2600 ms) with 4 mm thick axial and sagittal sections. Axial sections were acquired parallel to the plane connecting the anterior and posterior commissures (AC-PC lines).

The T2* DSC-MRI was performed utilizing a 2D single shot multislice Gradient Echo Echo Planar Imaging (GREEPI) sequence (TR/TE/FA: 1500 ms/40 ms/30°, BW: 2442 Hz/pixel, Echo spacing: 0.47 ms and Echo Planar Imaging (EPI) factor 64). Twenty consecutive slices of 4 mm slice thickness and 1.5 mm interslice gap with 50 dynamic acquisitions were obtained. Immediately after the end of the fifth dynamic acquisition, a bolus of 0.1 mmol/kg body weight of gadobutrol (Gadovist, Schering AG, Germany) was injected intravenously, at an injection rate of 4 mL/sec immediately followed by a bolus injection of 15 mL of saline at the same rate. Postcontrast 3D T1-w sequence (MPRAGE) was obtained after the acquisition of the perfusion data.

Postprocessing of the perfusion data was performed online using a dedicated software provided by the manufacturer. The arterial input function was calculated by manually defining a major artery (usually MCA) and parametric maps of relative CBV, CBF, and MTT values were automatically created. CBV, CBF, and MTT values of NAWM and NADGM areas were calculated by using 20 regions of interest (ROIs) located at the periventricular WM, semioval center, subcortical WM (in the frontal, parietal, temporal, and occipital lobes), thalami, putamen, and caudate nuclei, bilaterally, and also at the splenium and genu of the corpus callosum (CC). In order to optimize reproducibility, three CBV, CBF, and MTT measurements were obtained from each of the different NAWM and NADGM areas, which were then averaged. Two measurements were obtained from each caudate nucleus, due to its small size. All ROIs were fixed in size (radius of 2 mm) and were placed at the bolus peak of the GREEPI images, which show the vessels to better advantage and thus vascular structures were excluded. From the GREEPI images, ROIs were automatically transferred to the CBV, CBF, and MTT maps, using dedicated software (MRIcro Medical Analysis Viewer). In order to compare between different subjects, the calculated relative CBF, MTT, and CBV values were normalized for each patient with respect to the average values of CBF and MTT of the dentate gyrus of the hippocampi (set at 45 mL/100 mL/min and 4 secs resp.), a region assumed not to be affected in early MS [5].

### 2.5. Statistical Analysis

Group comparisons on demographic, clinical, and neuropsychological data were performed via one-way ANOVAs or Pearson chi-square tests for proportions when appropriate (evaluated at *α* = 0.05, two tailed). Group differences on regional perfusion values were assessed using one-way ANOVAs, separately for each ROI. Associations between perfusion measures and performance indices were assessed through Pearson correlation coefficients. All tests were evaluated at a Bonferroni-adjusted *α* = 0.05/20 (ROIs) = 0.0025. Corresponding partial correlations (separately) controlling for depression, anxiety, fatigue, EDSS, and illness duration were also computed.

In order to identify regions where individual variability in perfusion was specifically related to executive functions, we computed differences between perfusion performance correlation coefficients (simple minus inhibition and simple minus set shifting conditions, resp.), using Fisher's *z* transform. Executive function-specific associations between performance and perfusion metrics were established for a particular ROI upon meeting the following statistical criteria: (a) the zero-order correlation coefficient between performance (error rate or RT) and at least one perfusion metric (CBF or CBV) on the condition that involved executive processes was significant at alpha = .0025, (b) the corresponding coefficient on the nonexecutive condition did not reach significance at alpha = .05, and (c) the zero-order coefficient difference between corresponding “simple” and “executive” conditions was significant at a level adjusted according to the number of significant zero-order correlations for a particular condition (using the Bonferroni method).

## 3. Results

### 3.1. Demographic Data

The cohort of CIS patients included 17 women and 13 men with a mean age (±SD) of 31.4 ± 9.6 years. The neuropsychology comparison group included 25 women and 18 men with a mean age of 33.6 ± 10.2 years (*P* > .1; see Table 1).

### 3.2. Motor-Executive Measures

CIS patients showed significantly elevated reaction times (RTs) on both tasks and task conditions (with the exception of simple blocks in the Complex Decision task) compared to healthy controls, without evidence of reduced performance accuracy (Table 2).

### 3.3. Associations between Perfusion and Motor-Executive Measures

CIS patients had significantly elevated CBV and MTT values across all 20 ROIs and decreased CBF values in both caudate and left thalamus, compared to healthy controls. Several coefficients exceeded the Bonferroni-adjusted cutoff of *r* = .50 (*P* = .0025), all reflecting associations between CBV/CBF and performance on the inhibition and set shifting conditions. In all cases, the globally increased CBV values were associated with reduced performance: higher error rates and longer RTs. None of the coefficients with error rates or RT in the simple conditions of each task exceeded *r* = .22 (*P* = .12). Associations satisfying all three criteria presumed to reflect condition specificity of the observed associations are reported below.

**Figure 1:**
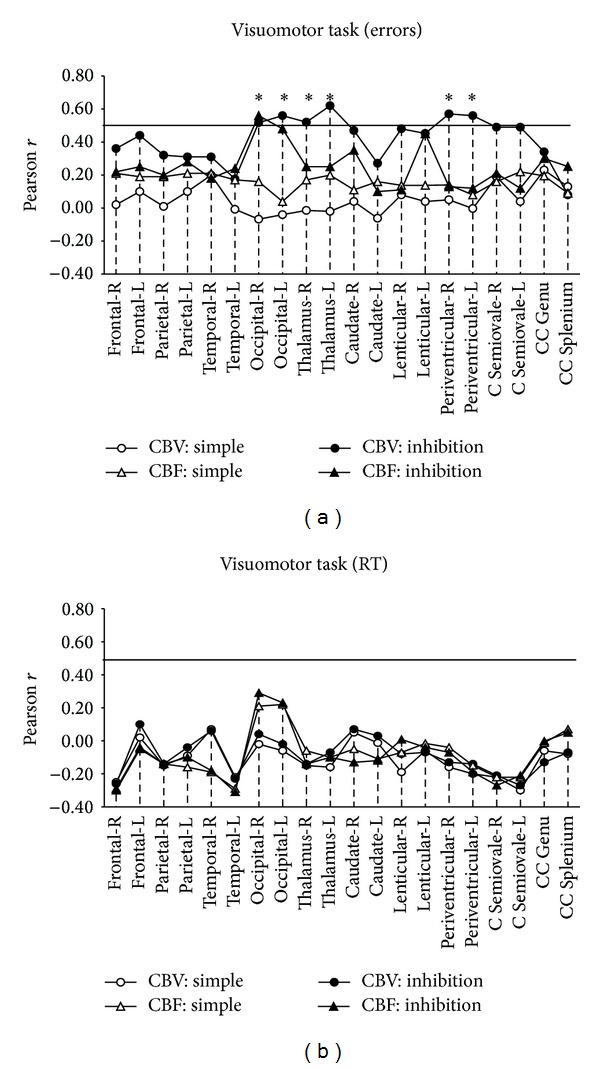
Correlation between performance accuracy and RT during the visuomotor learning task and CBV/CBF values for CIS patients. Zero-order Pearson correlation coefficients between performance accuracy (a) and RT (b) during the visuomotor learning task and CBF/CBV in 20 ROIs in the left (L) and right (R) hemispheres and the corpus callosum. Coefficients > 0.50 were significant at a Bonferroni-corrected alpha level of .0025 (indicated by horizontal solid lines). Asterisks indicate significant correlations which were also found to be condition specific (based on Fisher's *z* test for the difference between correlation coefficients in the “simple” and “executive” conditions).

*Visuomotor Learning Task*. Significant, positive correlation coefficients between error rates on the inhibition condition and CBV values were found in the following six ROIs: thalami, occipital, and periventricular NAWM, bilaterally (ranging between *r* = .52 and *r* = .62) (Figure 1(a)). Fisher's *z* tests confirmed that each of these ROIs correlations was significantly stronger in the inhibition as compared to the simple response condition (the critical value for 6 comparisons was set at *z* = 2.4, alpha = .05/6 = .008) (Table 3). Notably, none of the coefficients between perfusion and performance on the simple condition reached significance (*r* values ranged between −.04 and .22). Similarly, across conditions, correlations between perfusion and RT failed to reach significance (*r* ranged between −.32 and .29 (Table 3, Figure 1(b)). Thus, results indicated that regionally increased blood volume was associated with increased error rates when the task required inhibition of a previously learned stimulus-response association.

**Figure 2:**
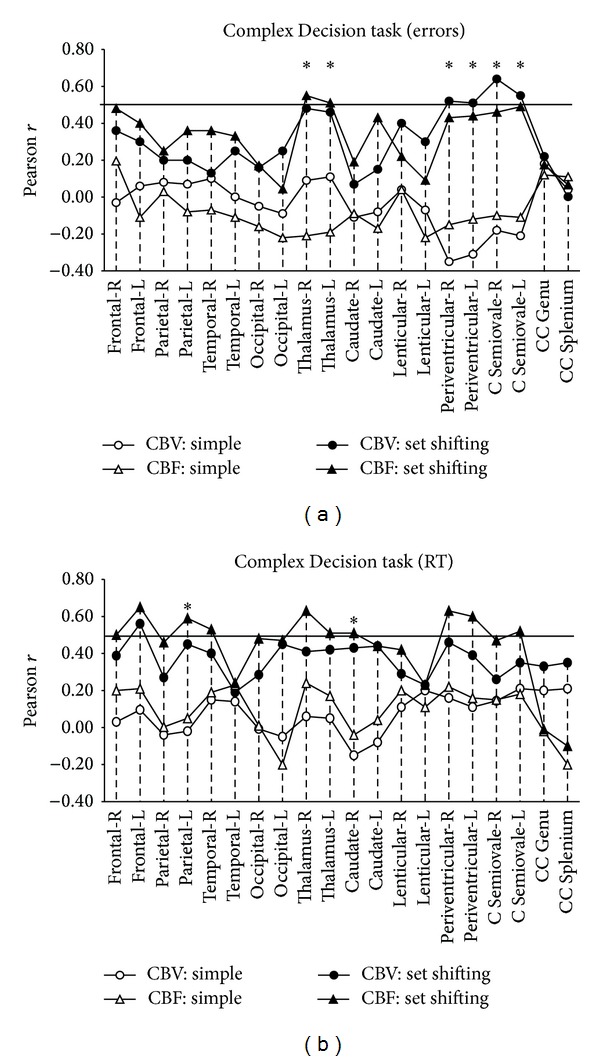
Correlation between performance accuracy and RT during the Complex Decision task and CBF/CBV values for CIS patients. Zero-order Pearson correlation coefficients between performance accuracy (a) and RT (b) during the Complex Decision task and CBF/CBV in 20 ROIs in the left (L) and right (R) hemispheres and the corpus callosum. Coefficients > 0.50 were significant at a Bonferroni-corrected alpha level of .0025 (indicated by horizontal solid lines). Asterisks indicate significant correlations which were also found to be condition specific (based on Fisher's *z* test for the difference between correlation coefficients in the “simple” and “executive” conditions).

*Complex Decision Task*. Significant positive associations which appeared to be specific to executive task requirements (set shifting ability) were found for the following perfusion metrics: (a) between CBV in the semioval center and periventricular NAWM (bilaterally) and error rates (Pearson correlations in the set shifting condition ranged between *r* = .51 and *r* = .64 (Figure 2(a))—as shown in Table 3, correlation differences between the simple and set shifting conditions ranged between −.76 and −.85 (*z* > 2.97, *P* < .003), (b) between CBF in the right (*r* = .55) and left thalamus (*r* = .51) and error rates (*z* > 2.77, *P* < .005), and (c) between CBF in the left parietal NAWM (*r* = .59) and right caudate (*r* = .51) and RT (*z* > 2.25, *P* < .024) (Figure 2(b)). None of the coefficients between perfusion and performance (error rates or RT) on the simple condition reached significance (Pearson *r* values ranged between −.38 and .23).

Including depression, anxiety, fatigue, EDSS and illness duration did not significantly affect the magnitude of correlation coefficients between performance and perfusion measures.

## 4. Discussion

The current study provides evidence on the functional significance of regional perfusion changes in NAWM and NADGM in patients with CIS. Performance perfusion associations were obtained for experimental tasks designed to isolate effects linked to psychomotor speed from effects linked to the engagement of brain circuits responsible for executive functions (inhibition and (strategy) set shifting). On average, CIS patients displayed significantly increased RTs on all but the constant condition of the Complex Decision task, in agreement with the existing literature on delayed processing speed even in the earliest stages of MS [15–17]. Notably, even when performance required executive processes (inhibition or strategy switching) CIS patients as a group did not commit errors at a higher rate than the group of gender-, age-, education-, and IQ-matched healthy controls.

In a recent study, we revealed globally increased CBV and MTT values, in NAWM and NADGM structures, and reduced CBF in some NADGM structures of 30 CIS patients compared with 30 age- and gender-matched healthy controls [5]. As the elevated regional CBV values are thought to reflect disease-induced inflammation [32, 33], it was suggested that postinflammatory vasodilation or even angiogenesis, that occur at the early stages of MS [34], leads to globally increased CBV. These findings agree with the recently reported global inflammation in patients with early MS [21]. As the disease progresses to CDMS the chronic perivascular inflammation leads to vascular damage [12] and global ischemia [5, 6, 8, 11, 35], which are associated with decreased CBF. Local inflammation appears to coincide or even precede demyelination and axonal loss [36] and may, therefore, be considered as a marker of disturbance in regional brain function (in the case of NADGM) and in local or long-range connectivity (in the case of NAWM).

The novel finding in the present study is that both error rate and RTs were positively related to CBV and/or CBF values in several NAWM and NADGM ROIs. Perfusion disturbances in the thalami, caudate, periventricular NAWM, and the semioval center appeared to be of crucial importance in determining executive performance given that significant, condition-specific associations were found on both tasks. While periventricular WM contains important tracts connecting the prefrontal cortex with other cortical and subcortical regions, including the basal ganglia and thalamic nuclei [37], evidence linking physiological abnormalities in these regions and executive performance has been sparse. For instance, Smith et al. [38] found significant correlations between WM hyperintensity in periventricular regions and performance on the executive factor of a battery of neuropsychological tests in a large cohort of patients diagnosed with mild cognitive impairment. Moreover, the degree of leukoaraiosis in periventricular WM and the semioval center was a potent predictor of executive performance among patients with subcortical vascular dementia [39]. Li et al. [40] recently reported significant correlations between measures of WM integrity (mean diffusivity, fractional anisotropy, and N-acetylaspartate/creatine) and executive function measures (mainly set shifting ability as indicated by the Trail Making Test) in patients presenting with ischemic leukoaraiosis. Similarly, fractional anisotropy in the thalamus emerged as key correlate of executive task performance among trauma brain injury (TBI) patients [41].

It is important to note that neuroimaging findings in patients with widespread, but not necessarily uniform, brain pathology are not expected to bear significant brain-behavior associations for the entire set of component regions involved in a particular type of function (executive in the current case). Rather, perfusion-function associations obtained in the current study are more likely to point out components of the brain mechanisms for executive functions which are predominantly impaired in the early stages of demyelinating disease.

In conclusion, the findings reported here corroborate our hypothesis that regional perfusion changes, probably associated with active inflammatory processes in brain tissue, may be detrimental for simple executive functions, including inhibition ability and cognitive flexibility, in patients with CIS. Moreover, the observed differences in the magnitude of correlations between perfusion indices and task conditions requiring simple visuomotor processing and conditions involving executive functions (inhibition or set shifting) permitted the identification of those sections of NAWM and/or NADGM structures, such as the thalami and periventricular white matter, where perfusion interferes with the brain circuits responsible for these executive functions.

## Figures and Tables

**Table 1:** Clinical, demographic, and neuropsychological information on the patients and healthy controls when appropriate (mean ± SD and range in parentheses).

	CIS (*n* = 30)	Controls (*n* = 43)
Gender:		
Men	14	25
Women	16	18
Handedness:		
Right	27	37
Left	3	6
First symptom:		
Optic neuritis	6	—
Brainstem-cerebellar	11	
Spinal	1	
Sensorimotor	6	
Polysymptomatic	6	
Age (years)	31.4 ± 9.6 (15–51)	33.6 ± 10.2 (16–54)
Education (years)	13.6 ± 2.6 (6–18)	14.4 ± 3.2 (6–19)
Illness duration (years)	1.5 ± 1.5 (0–5)	—
EDSS	1.0 ± .5 (0–2)	—
WASI Vocabulary (z)	.80 ± 1.0 (−1.5 to 2.5)	.35 ± 0.9 (−1.5 to 2.1)
WASI Matrices (z)	−.25 ± .9 (−2.5 to 1.4)	−.08 ± .8 (−2.2 to 1.6)
CESD	13.0 ± 12.7 (1–49)	—
STAI-B (Trait Anxiety)	44.2 ± 10.6 (26–68)	—
Fatigue	29.7 ± 11.2 (10–50)	—

EDSS: Expanded Disability Status Scale; CESD: Center for Epidemiological Studies Depression Scale; STAI-A: State-Trait Anxiety Inventory Form Y; Fatigue: Fatigue Severity Scale; WASI: Wechsler Abbreviated Scale of Intelligence. Standard (*z*) scores on neuropsychological tests.

**Table 2:** Descriptive statistics for motor-executive task performance.

	CIS (*n* = 30)	Controls (*n* = 43)
	M ± SD	M ± SD
Visuomotor learning task		
Simple trials-RT (ms)**	744 ± 264	599 ± 137
Inhibition trials-RT (ms)**	960 ± 254	842 ± 205
Simple trials-% errors	2.4 ± 3.5	3.1 ± 4.4
Inhibition trials-% errors	6.0 ± 5.6	6.4 ± 6.2
Complex Decision task		
Simple trials-RT (ms)	676 ± 146	668 ± 214
Set shifting trials-RT (ms)**	917 ± 241	763 ± 255
Simple trials-% errors	15.2 ± 9.3	15.9 ± 9.2
Set shifting trials-% errors	12.4 ± 8.6	13.7 ± 9.0

Group differences: *P < .01, **P < .001. Raw data for comparison are presented here from a subgroup of 43 controls chosen from the normative sample, matched on gender, age, education, FSIQ, and place of residence (urban versus rural) to the group of CIS patients.

**Table 3:** Correlation differences between simple and inhibition trials and between simple and setshifting trials in the visuomotor and Complex Decision tasks, respectively.

	Visuomotor learning task error rate RT				Complex Decision task error rate RT			
	CBV	CBF	CBV	CBF	CBV	CBF	CBV	CBF
Frontal-R	−.34	−.01	−.29	−.30	−.39	−.29	−.36	−.30
Frontal-L	−.34	−.06	−.04	−.05	−.24	−.51	−.47	−.44
Parietal-R	−.31	−.01	−.14	−.14	−.12	−.22	−.31	−.46
Parietal-L	−.21	−.07	−.16	−.10	−.13	−.44	−.47	−.54∗
Temporal-R	−.11	.03	−.19	−.18	−.03	−.43	−.25	−.34
Temporal-L	−.18	−.07	−.29	−.31	−.25	−.44	−.05	−.01
Occipital-R	−.60*	−.40	.21	.29	−.21	−.33	−.29	−.47
Occipital-L	−.61*	−.44	.22	.23	−.34	−.26	−.50	−.67
Thalamus-R	−.53*	−.08	−.06	−.14	−.39	−.76**	−.35	−.39
Thalamus-L	−.64*	−.05	−.10	−.10	−.35	−.70**	−.37	−.34
Caudate-R	−.43	−.24	−.05	−.13	−.18	−.28	−.58	−.55*
Caudate-L	−.33	.06	−.11	−.12	−.23	−.60	−.52	−.40
Lenticular-R	−.44	.03	−.07	.01	−.36	−.18	−.18	−.22
Lenticular-L	−.41	−.31	−.02	−.04	−.37	−.31	−.03	−.12
Periventricular-R	−.53*	.01	−.04	−.07	−.85**	−.58	−.30	−.41
Periventricular-L	−.56*	−.04	−.15	−.19	−.79**	−.56	−.28	−.44
C semiovale-R	−.30	−.05	−.22	−.27	−.82**	−.56	−.12	−.32
C semiovale-L	−.45	.10	−.22	−.21	−.76**	−.60	−.14	−.34
CC genu	−.11	−.11	−.01	−.01	−.04	−.05	−.13	−.01
CC splenium	.05	−.16	.07	.05	.04	.04	−.14	−.10

*Correlation differences associated with significant Fisher's *z* values using Bonferroni correction (according to the number of significant zero-order correlations in each condition). Only correlation differences that satisfy all three conditions of condition specificity (see Section 2.5) are marked with asterisks. Negative correlation differences imply greater degree of association between perfusion metrics and performance on the more demanding condition of each task.
